# Activating Thermoplastic Polyurethane Surfaces with Poly(ethylene glycol)-Based Recombinant Human α-Defensin 5 Monolayers for Antibiofilm Activity

**DOI:** 10.1021/acsabm.4c00732

**Published:** 2025-02-20

**Authors:** Xavier Rodríguez Rodríguez, Adrià López-Cano, Karla Mayolo-Deloisa, Oscar Q. Pich, Paula Bierge, Nora Ventosa, Cristina García-de-la-Maria, José M. Miró, Oriol Gasch, Jaume Veciana, Judith Guasch, Anna Arís, Elena Garcia-Fruitós, Imma Ratera, the FUNCATH investigators

**Affiliations:** 1 Institute of Materials Science of Barcelona (ICMAB-CSIC), Campus UAB, Bellaterra 08193, Spain; 2 Networking Research Center on Bioengineering, Biomaterials and Nanomedicine (CIBER-BBN), Campus UAB, Bellaterra 08193, Spain; 3 IRTA, Ruminant Production, Torre Marimon, 08140 Caldes de Montbui, Barcelona, Spain; 4 Tecnologico de Monterrey, Institute for Obesity Research, School of Engineering and Sciences, Av. Eugenio Garza Sada 2001, 64849 Monterrey, Nuevo León, México; 5 Laboratori de Recerca en Microbiologia i Malalties Infeccioses, Hospital Universitari Parc Taulí, Institut d’Investigació i Innovació Parc Taulí (I3PT-CERCA), Universitat Autònoma de Barcelona, 08208 Sabadell, Spain; 6 Institut de Biotecnologia i Biomedicina, Universitat de Barcelona, 08193 Bellaterra, Spain; 7 Infectious Diseases Service, Hospital Clinic-FCRB-IDIBAPS, Universitat de Barcelona, 08036 Barcelona, Spain; 8 Infectious Diseases Biomedical Research Networking Center (CIBERINFEC), Instituto de Salud Carlos III, 28029 Madrid, Spain; 9 Servei de Malalties Infeccioses, Hospital Universitari Parc Taulí, Institut d’Investigació i Innovació Parc Taulí (I3PT-CERCA), Universitat Autònoma de Barcelona, 08208 Sabadell, Spain; 10 Dynamic Biomimetics for Cancer Immunotherapy, Max Planck Partner Group, ICMAB-CSIC, Campus UAB, Bellaterra 08193, Spain; 11 FUNCATH Investigators are indicated in the author contributions

**Keywords:** Antimicrobial surfaces, Biofilm inhibition, Thermoplastic polyurethane, TPU, Assembled monolayers, Click reaction, Host defense peptides, HDP, Gram-positive and Gram-negative bacteria

## Abstract

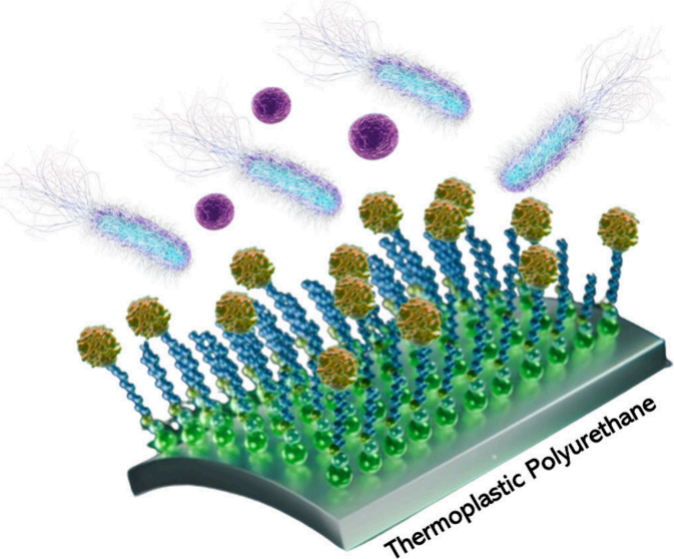

Addressing multidrug-resistant microbial infections linked to implantable biomedical devices is an urgent need. In recent years, there has been an active exploration of different surface coatings to prevent and combat drug-resistant microbes. In this research, we present a facile chemical modification of thermoplastic polyurethane (TPU) surfaces with poly(ethylene glycol)-based recombinant human α-defensin 5 (HD5) protein with antimicrobial activity. TPU is one of the most relevant materials used for medical devices with good mechanical properties but also good chemical resistance, which makes it difficult to modify. The chemical modification of TPU surfaces is achieved via a three-step procedure based on (i) TPU activation using hexamethylene diisocyanate (HDI); (ii) interfacial reaction with poly(ethylene glycol) (PEG) derivatives; and finally, (iii) a facile click reaction between the PEG-maleimide terminated assembled monolayers on the TPU and the cysteine (-thiol) termination of the recently designed recombinant human α-defensin 5 (HD5) protein. The obtained PEG based HD5 assembled monolayers on TPU were characterized using a surface science multitechnique approach including scanning electron microscopy, atomic force microscopy, contact angle, and X-ray photoelectron spectroscopy. The modified TPU surfaces with the HD5 protein derivative exhibit broad-spectrum antibacterial properties reducing biofilm formation against *Pseudomonas aeruginosa* (Gram-negative), methicillin-resistant *Staphylococcus aureus* (MRSA) (Gram-positive) and methicillin-resistant *Staphylococcus epidermidis* (MRSE) (Gram-positive). These findings underscore the substantial potential of protein-modified TPU surfaces for applications in combating bacterial infections associated with implantable materials and devices.

## 1 Introduction

Over the last 50 years, the utilization of implantable biomaterials and medical devices like catheters has played a crucial role in treating diseases. However, the use of such devices has been linked to bacterial infections, which usually need to be managed with withdrawal of the device, prolonged antibiotic therapies, and longer hospital admissions. This situation imposes a substantial economic burden on both the government and society.^1−6^ Healthcare-associated infections (HAIs) are related with high morbidity and mortality and excess costs in care of patients^7^ with over 60% attributed to medical devices.^8^ According to research, over 2.5 million HAI episodes occur every year in Europe with more than 90,000 deaths attributed to the 6 most common types: healthcare-associated pneumonia, health care-associated urinary tract infection (UTIs), surgical site infection, health care-associated *Clostridium difficile*infection, healthcare-associated neonatal sepsis, and healthcare-associated bloodstream infection.^9^ According to a study involving 15 European countries, one of the most frequently isolated microorganisms from patients suffering HAIs are bacteria such as *Pseudomonas aeruginosa*, coagulase-negative *Staphylococcus* spp., and *Escherichia coli*.^10^

Despite the widespread use of antibiotics for treating bacterial infections, their overuse and misuse during the decades have accelerated the emergence and spread of multidrug-resistant (MDR) bacteria in clinical settings.^11−16^ Conventional antibiotics demonstrate limited efficacy against MDR bacteria, which poses a severe threat to human life. Consequently, efforts have been directed toward exploring alternative strategies to combat bacterial infections, such as incorporating antibacterial modifications on materials.^17−26^

Biofilm is the primary form of surface bacterial contamination, which causes serious problems and can easily lead to drug resistance. In the biofilm, bacterial cells can exhibit up to a 100-fold increase in antimicrobial resistance (AMR) when compared to planktonic cells.^27^ In fact, in more than 60% of cases UTIs are associated with biofilm formation on the surface of catheters.^28^ Surface antibacterial methods can be categorized into repelling or contact-killing surfaces. The widely investigated release-killing reagents in modified surface modification of materials mainly contain inorganic metal particles, antibiotics, or antibacterial enzymes. The conventional contact-killing materials for antibacterial materials surfaces mainly include quaternary ammonium salts, antimicrobial peptides (AMPs), and *N*-halamines.^29^

Host Defense Peptides (HDPs), are short AMPs with a conserved sequence and structure produced by the innate immune system of organisms of all life kingdoms. These peptides have a potent and broad-spectrum microbiocidal activity because of their cationic nature and hydrophobicity.^27^ In addition, their multiple modes of action allow a very low induction of resistance compared to the traditional antimicrobials.^30^ We have previously developed a new generation of tailored antimicrobials, using a rational design of recombinant multidomain proteins based on HDPs, demonstrating their great potential against HAI-causing bacteria in either biofilm or planktonic forms.^31^ HDPs are an interesting alternative because they are effective against a broad spectrum of bacteria, while presenting low toxicity to mammalian cells.^31^ Moreover, recently developed surface biofunctionalization strategies^32−37^ have been used to covalently anchor such novel recombinant proteins on gold model surfaces obtaining effective antibiofilm surfaces.^38^

Thermoplastic polyurethane (TPU) based catheters (Figure S1) represent an indispensable class of implantable biomaterials used in hospitals for the transfer of body fluids or the administration of medication. As far as developing antibacterial TPUs is concerned, various active moieties have already been incorporated along with a segmented polyurethane (Figure S1) such as antibiotics like chloramphenicol or metallic (cobalt or silver) nanoparticles,^39^ but all of them present important concerns regarding antibiotic resistance or toxicity. For this reason, it is urgent to find alternatives to antibiotics and metals, such as silver, to avoid nosocomial infections on TPU surfaces. TPU surfaces can be activated by using different strategies like ultraviolet irradiation, gamma irradiation, and interfacial modification.^40^ The process of activation improves the TPU surfaces’ functionality;^41,42^ however, it is still challenging to chemically modify TPU surfaces due to its chemical resistant properties.

**Scheme 1:**
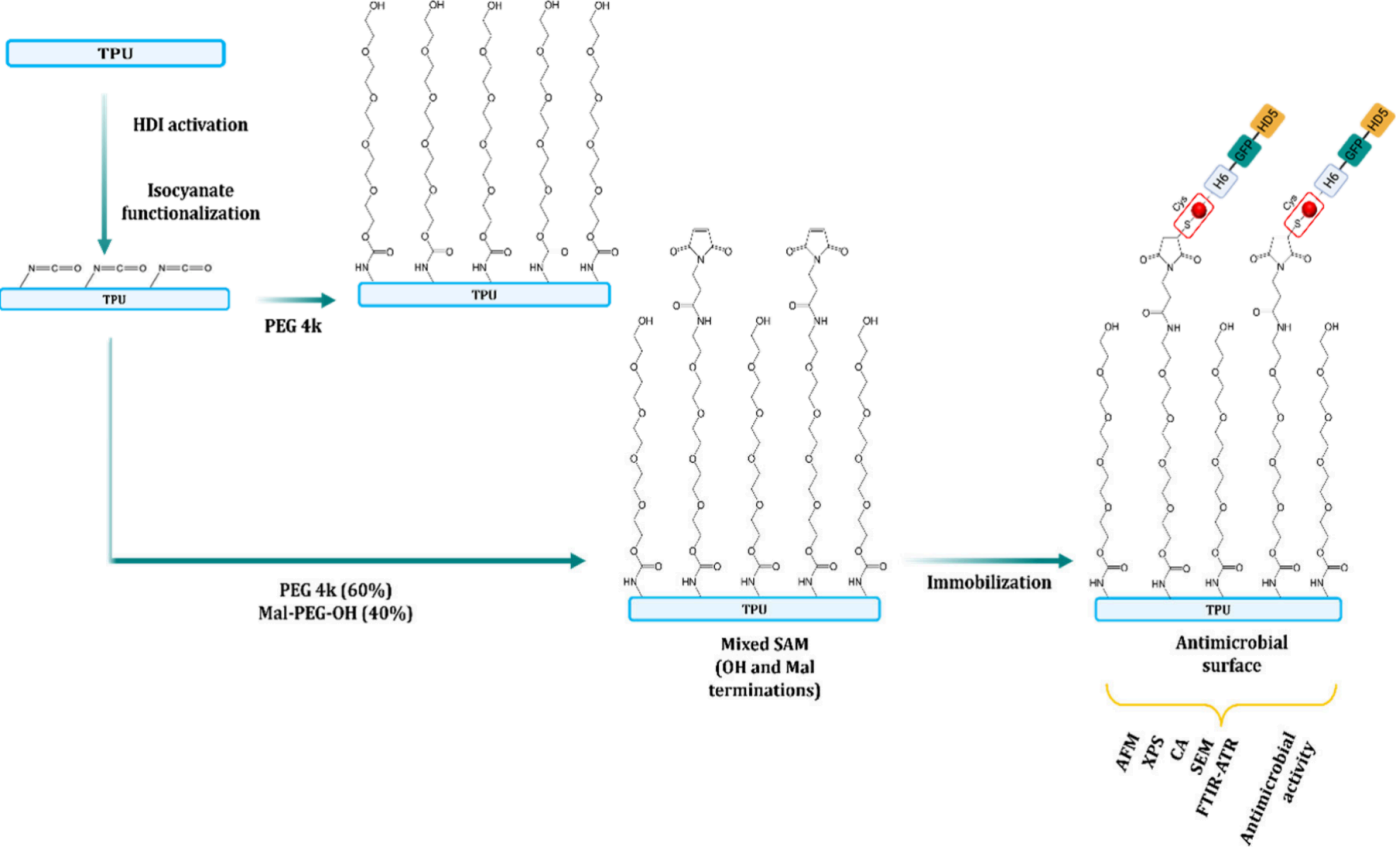
General Strategy Used for the Chemical Activation and Immobilization of HD5-GFP-H6-Cys on TPU Surfaces Using a Bioclickable Surface-Induced Assembled Monolayer Strategy

In this study, we propose a controlled design and synthesis of antibiofilm surfaces by coating medical grade TPU surfaces with recombinant HDPs to confer broad-spectrum antibacterial properties on medically relevant TPU surfaces. The chemical modification of TPU surfaces is achieved via a three-step procedure based on activation of the surface using hexamethylene diisocyanate (HDI) followed by an interfacial reaction with poly(ethylene glycol) (PEG) derivatives with a maleimide terminal group forming a surface-induced mixed assembly. PEG derivatives are used for grafting because they are known to enhance hydration effects that prevent bacterial attachment. High-density PEG layers will be obtained to resist nonspecific protein adsorption, which is often a precursor to bacterial attachment. Finally, a click reaction between the PEG-maleimide termination on the TPU and the cysteine (-thiol) terminated HD5 (HD5-GFP-H6-Cys) antimicrobial protein takes place to generate the anti-biofilm surface. The use of multiple, complementary analytical techniques to study the properties and behavior of a surface yield more detailed and reliable information about its chemical, physical, and structural properties which is especially important to prove the successful functionalization of the TPU. Thus, a multitechnique approach using atomic force microscopy (AFM), X-ray photoelectron spectroscopy (XPS), scanning electron microscope (SEM), water contact angle (WCA), Fourier transform infrared spectroscopy–attenuated total reflectance (FTIR-ATR), and fluorescent plate reader was used to perform a physicochemical characterization of the surfaces (Scheme 1).

## 2 Results and Discussion

After preparation of TPU discs through an extrusion process from pellets, the surface modification of the TPU discs was done in a three-step procedure. In the first step, the TPU films were functionalized with HDI. In the second step, PEG4K for the control surface-induced assembled monolayer (TPU-PEG) and 60% PEG4K and 40% Mal-PEG5K-OH for the surface-induced mixed assembled monolayer (TPU-PEG-Mal) were grafted onto the TPU surface to obtain the functionalized TPU. The length of the maleimide tail was longer than the PEG one to increase the availability of the reactive unit toward the click reaction with the protein. Specifically, TPU films were immersed in 10% (w/v) solutions of 100% PEG4K or mixed 40% Mal-PEG5K-OH and 60% PEG4K and left to react overnight at 45 °C, letting the hydroxyl groups react with the isocyanate ends groups of the activated TPU surface.

In the last step, the protein HD5-GFP-H6-Cys was anchored to the prefunctionalized TPU surface via a click Diels–Alder reaction between the thiol of the cysteine group of the protein and the maleimide terminated group of the mixed assembled monolayer (Scheme 1).

**Figure 1:**
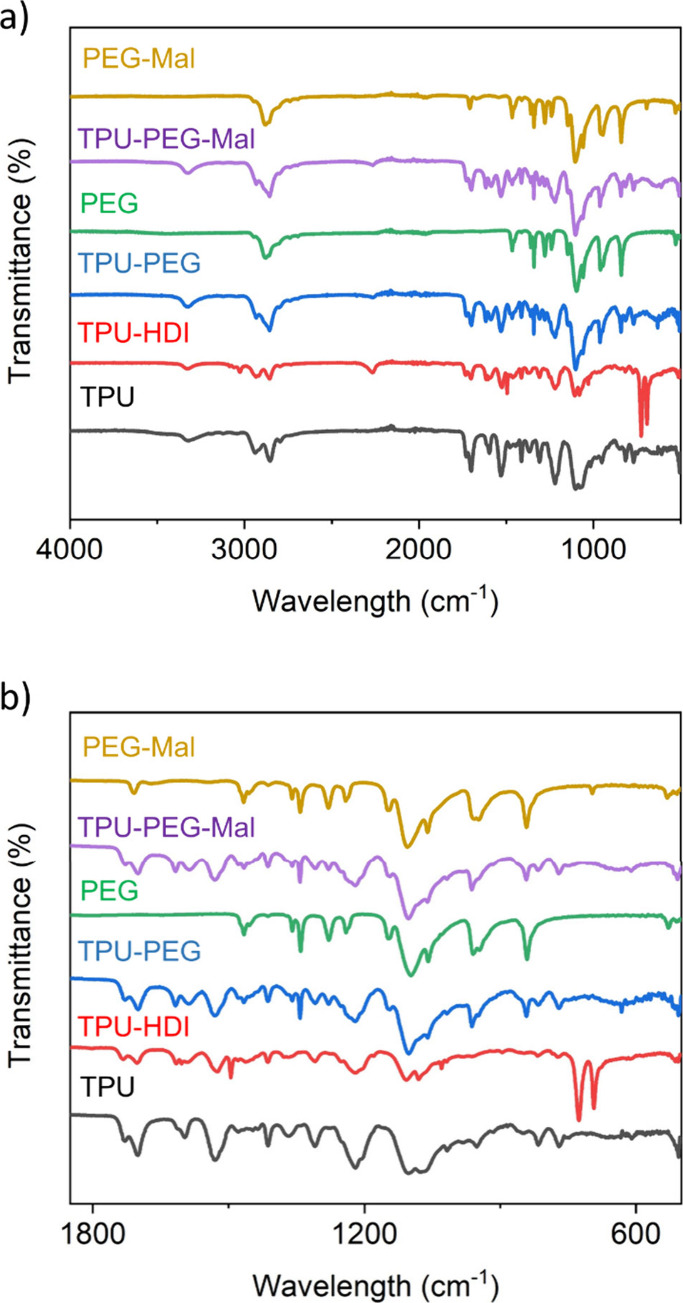
a) FTIR spectra: (black) unmodified thermoplastic polyurethane, (red) functionalization of TPU with HDI, (blue) TPU with a PEG4K assembled monolayer (TPU-PEG), (green) PEG4K, (purple) TPU with a mixed assembled monolayer (40% PEG4K and 60% Mal-PEG5K-OH) (TPU-PEG-Mal), and (yellow) Mal-PEG5K-OH. b) Zoomed-in view of the spectra in panel a.

Then, a multitechnique approach was used to characterize the resulting modified surfaces. FTIR, XPS, WCA, and fluorescence plate reader were used to characterize the chemical modification of the surface. The discs of nonmodified TPU contain NH and C=O groups (Figure S1); thus, its FTIR spectrum shows two defined peaks corresponding to the stretching vibrations of these groups, one at ∼3400 cm^–1^ (NH) and the other at ∼1650 cm^–1^ (C=O). For the activated TPU-HDI surface, a new band that was not present on the unmodified TPU appears at 2250 cm^–1^ which is indicative of the successful activation of the TPU surface with the isocyanate molecules. Moreover, in the second step of the functionalization, this peak clearly disappeared, indicating that the reaction of the isocyanate groups with the hydroxyl groups of the PEG molecules was effectively achieved. Additionally, the PEG modification of the surface originates peaks at 1343 and 1466 cm^–1^ which correspond to the bending vibration of −CH_2_ and −CH_3_ and at 842 and 957 cm^–1^ that are attributed to the bending vibrations of −CH_2_CH_2_O– and −COC–, respectively. The high intensity of the FTIR peaks attributed to the PEG derivatives are indicative of the desired high density of the PEG monolayer assembly on the TPU. The FTIR analysis indicates the successful modification of TPU surfaces based on the presence of the characteristic peaks expected for the compounds used for the TPU functionalization (Figures 1 and S2–S4).

**Figure 2:**
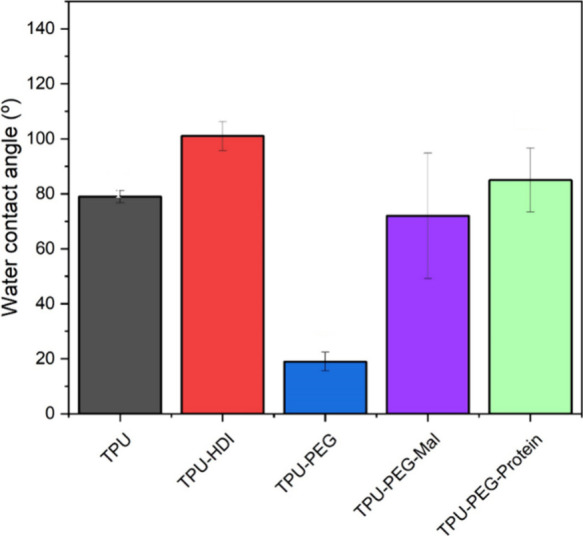
Water contact angle of (black) unmodified TPU, (red) TPU-HDI, (blue) TPU-PEG, (purple) TPU-PEG-Mal, and (green) protein anchored to TPU-PEG-Mal substrate (TPU-PEG-Protein).

The WCA of the unmodified TPU substrate was 79°. Its increase to 101° after the modification with HDI and its decrease to 19° when the hydrophilic PEG was anchored to it are clear indications of the successful functionalization steps of the process. The low WCA for TPU-PEG is also an indication of the high density of the surface-induced PEG assembly. For the mixed molecular assembly (TPU-PEG-Mal) and the final protein functionalized surfaces, the WCA increases again to 72° and 85°, indicating its more hydrophobic nature, as expected (Figure 2).

Moreover, WCA and FTIR of TPU-PEG surfaces have been measured during 24 and 48 h in water, PBS, and cell culture media (RPMI) confirming the robustness, stability, and high density of the PEG anchored on the TPU surfaces in different media relevant for its final applications (Figure S2–S5) due to the covalent chemical functionalization of TPU.

**Figure 3:**
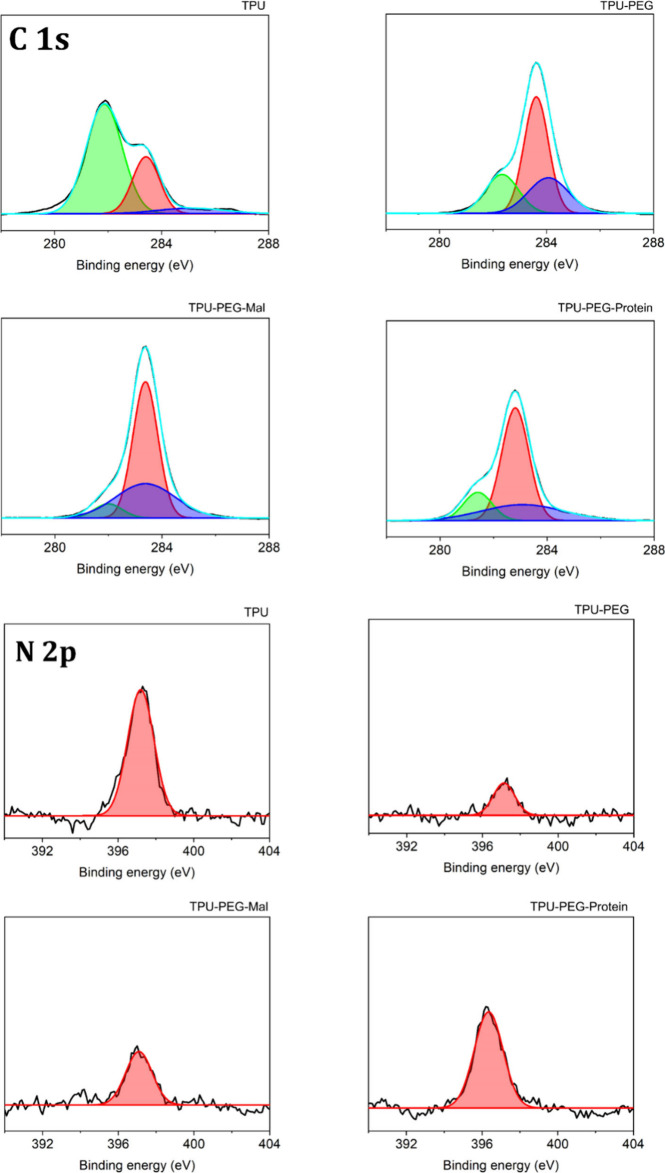
XPS spectra of C 1s and N 2p for unmodified TPU, TPU-PEG, TPU-PEG-Mal, and TPU-PEG-Protein.

The TPU substrates were further characterized by using high-resolution XPS spectra to determine the nature and the level of functionalization present on the surface in each synthetic step. Table 1 summarizes the percentage of elements present on the TPU modified surfaces. The elemental composition (carbon, nitrogen, and oxygen) of the surfaces was calculated from the XPS spectra. Figures 3 and S6 show the different spectra obtained for the different samples of unmodified and modified TPU. The relative composition ratio based on the area of the deconvoluted peaks is shown in Table 1.

**Table 1:** Binding Energy (eV) and Relative Composition Ratio Based on the Area (%) of Each Peak for the Atomic Percentages Obtained from the XPS Spectra of the Different Samples Studied

TPU		TPU-PEG		TPU-PEG-Mal		TPU-PEG-Protein	
BE (eV)	composition ratio (%)	BE (eV)	composition ratio (%)	BE (eV)	composition ratio (%)	BE (eV)	composition ratio (%)
C 1s							
281.9	50.8	282.34	16.3	282	4.7	281.4	11.2
283.4	19.6	283.62	36.3	283.37	40.5	282.8	42.3
285.1	4.9	284.07	18.1	283.73	24.4	283.1	18.0
N 2p							
397.2	3.0	397.1	0.4	397.1	0.7	396.4	1.9
O 1s							
527.6	1.2	528.5	1.3	528.3	1.6	527.7	1.6
529.63	20.6	530.1	27.6	529.9	28.1	529.3	25.0

The C 1s peak is resolved in three components: (i) C–C and C–H at ∼281–282 eV; (ii) C–O–C and C–OH at ∼283 eV, and (iii) N–COO at ∼284–285 eV from the urethane and isocyanate termination. After the functionalization with the PEG and PEG-Mal molecules, an increase of the C–O–C and C–OH peaks and a decrease of the C–C and C–H peaks are observed, confirming the covalent anchoring of the PEG units. An increase of the N–COO peak is also observed after the modification of the TPU due to the HDI activation performed previously for the PEG reaction. The TPU-PEG-Protein surface shows a small decrease in the HNCOO due to the covering of the maleimide groups by the protein. The C–O–C/C–OH and C–C/C–H peaks slightly increase, which is attributed to the amino acids of the protein. Regarding the N 2p peak between ∼396–397 eV, the TPU-PEG substrate presents a considerable decrease due to the hindering of the nitrogen atom of the TPU by the PEG molecules. The N 2p peak follows an increase after the PEG-maleimide anchoring due to the N atoms of the maleimide, and a more intense increase is observed after the functionalization with the protein due to its high N content, demonstrating the successful functionalization of the TPU surface with the antimicrobial protein (Figure 3 and Table 1). Regarding the oxygen, two peaks are identified, ∼527–528 eV of the C=O and C–OH and ∼529–530 eV of the C–O–C and NCOO, which increase after the functionalization due to the incorporation of oxygen containing molecules like the PEG for TPU-PEG and TPU-PEG-Mal. The oxygen content decreases for the TPU-PEG-Protein due to the presence of the protein, which increases the number of carbon atoms with respect to oxygen (Figure S6).

**Figure 4:**
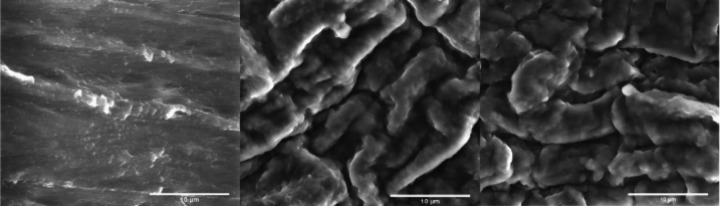
Representative SEM images of (left) unmodified TPU, (center) TPU functionalized with 100% of PEG4K surface-induced assembled monolayer, TPU-PEG; and (right) TPU functionalized with a mixed surface-induced assembled monolayer of 60% PEG4K and 40% Mal-PEG5K-OH, TPU-PEG-Mal. Scale bar: 10 μm.

**Figure 5:**
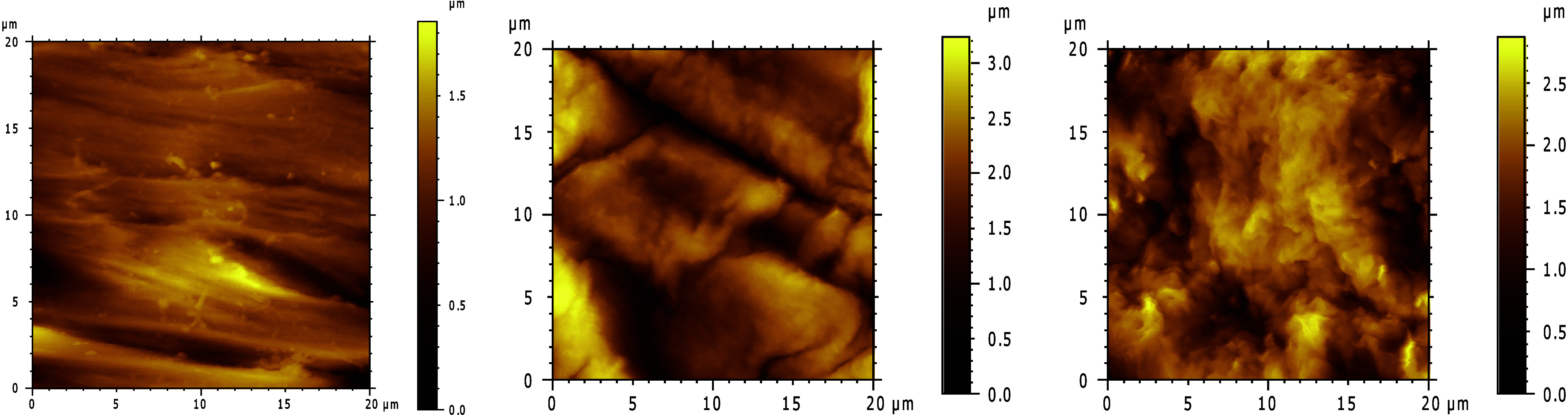
Representative AFM images of (left) unmodified TPU, (center) TPU functionalized with a 100% PEG4K surface-induced assembled monolayer, TPU-PEG; and (right) TPU functionalized with a mixed surface-induced assembled monolayer of 60% PEG4K and 40% Mal-PEG5K-OH, TPU-PEG-Mal.

Surface morphology and topography were evaluated by SEM and AFM. Representative SEM and AFM images of the TPU surfaces before and after each step of the modification are shown in Figures 4, 5, and S7. After imaging different positions of all the samples, a clear increase of the roughness of the samples is observed after the functionalization steps, for the TPU-PEG and TPU-PEG-Mal surfaces. Specifically, the roughness root-mean-square (RMS) increases from 0.375 μm for the unmodified TPU to 1.159 μm and 1.323 μm for the TPU-PEG and TPU-PEG-Mal, respectively. Such a roughness increase is also indicative that the TPU surface has been modified.

To visualize if the surface has been functionalized with the protein, the fluorescence was measured using a microplate reader (Figure S8) taking advantage of the green fluorescent protein (GFP) fused to the antimicrobial active moiety of the protein used, i.e., HD5-GFP-H6-Cys. From these images, it was observed that higher fluorescence emission is obtained using 10 μM protein concentration. Thus, for the click reaction, 10 μM protein was selected, and the reaction was incubated for 2 h in a wet chamber.

**Figure 6:**
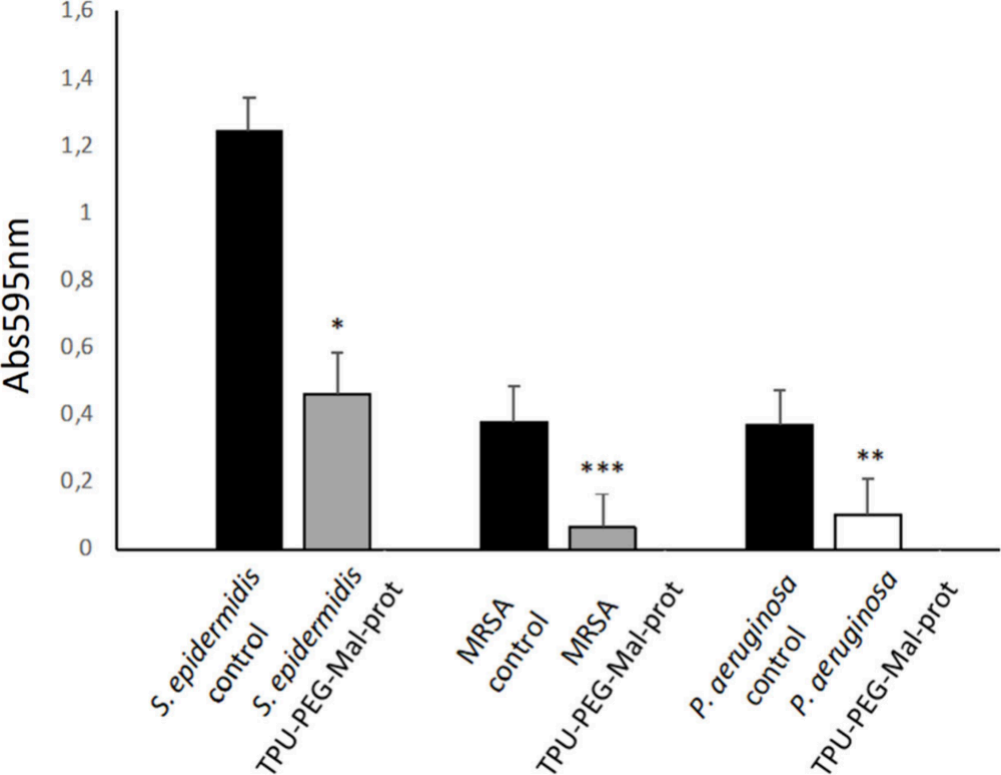
Inhibition of *Pseudomonas aeruginosa* (Gram-negative), methicillin-resistant *Staphylococcus aureus* (MRSA) (Gram-positive), and methicillin-resistant *Staphylococcus epidermidis* (MRSE) (Gram-positive) biofilms by HD5-GFP-H6-Cys protein anchored on the TPU-PEG-Mal surfaces (TPU-PEG-Mal-Prot). Asterisks depict significant differences compared to the control (*p-value ≤ 0.05, **p-value ≤ 0.005, ***p-value ≤ 0.0005).

Finally, the antimicrobial activity for inhibition of the formation of biofilm was assessed against three bacteria relevant in HAIs: *Pseudomonas aeruginosa* (Gram-negative), methicillin-resistant *Staphylococcus aureus* (MRSA, Gram-positive) and methicillin-resistant *Staphylococcus epidermidis* (MRSE, Gram-positive). Results demonstrated that TPU surfaces functionalized with antimicrobial protein HD5-GFP-H6-Cys avoid the formation of biofilm from 50% to 99% in the three different pathogens studied and involved in HAI, whereas nonfunctionalized TPU surfaces did not (Figure 6). Among the pathogens tested (*P. aeruginosa*, MRSA, and MRSE) there were Gram-negative and Gram-positive bacteria and AMR bacteria such as MRSA and MRSE, showing the versatility and broad-spectrum activity of the tested strategy.

## 3 Conclusions

Biofilm formation on medical device surfaces enhances antimicrobial resistance, emphasizing the need for innovative solutions. The use of AMPs, particularly HDPs, has emerged as a promising approach due to their broad-spectrum activity and low propensity for inducing resistance. This study demonstrates a novel method for modifying TPU surfaces, one of the materials most used for medical devices, with recently designed recombinant HDPs, offering broad-spectrum antibacterial properties. Specifically, a facile antibacterial modification of TPU surfaces has been achieved using an initial isocyanate activation of the chemically resistant TPU surface. Then, an interfacial reaction with PEG derivatives and surface-induced click reaction between the PEG-maleimide terminated assembled monolayer on the TPU and the cysteine (-thiol) terminated HD5 protein gives rise to TPU surfaces with broad-spectrum antibiofilm activity. Characterization using a multitechnique approach confirms the efficacy and stability of the antibiofilm surfaces. The surface modification strategy developed, together with the performance of the protein modified TPU surfaces reducing biofilm formation against *P. aeruginosa* (Gram-negative), methicillin-resistant MRSA (Gram-positive), and MRSE (Gram-positive), along with the cost-effective synthesis and the expected low induction of resistance of the protein, suggest its potential applications to fight surface-associated bacterial infections of medical devices like catheters or implants.

This research advances the development of customized antimicrobial surfaces, offering a significant alternative to traditional antibiotics and metals, crucial for tackling multidrug-resistant bacteria and enhancing patient outcomes in healthcare.

## 4 Materials and Methods

### 4.1 Materials

Dibutyltin dilaurate 95% (DD), hexamethylene diisocyanate 98% (HDI), poly(ethylene glycol) 4000 (PEG4K), and poly(ethylene glycol) maleimide (Mal-PEG5K-OH) were purchased from Merck. Thermoplastic polyurethane (TPU) beads were ordered from Lubrizol (Tecothane 2085A-B20 and Tecothane 1085A). Toluene was ordered from Chem-Lab.

### 4.2 TPU Disc Fabrication

TPU disc fabrication was based on an extrusion process of the TPU pellets of medical grade Tecothane (TT-1085A). These pellets were melted to form a TPU bar of 7.8 mm diameter, and then they were cut into 1 mm thick pieces. From them, discs of 7.5 mm diameter were cut with a punch.

### 4.3 HD5-GFP-H6-Cys Production

The HD5-GFP-H6-Cys was based on the mature sequences of HD5 fused to the green fluorescent protein (GFP) gene through the linker sequence (GGSSRSS) and C-terminally fused to a 6 histidine (H6)-tag for protein purification and a cysteine, as described in ref (31). The HD5-GFP-H6-Cys sequence was codon-optimized by GeneArt (GeneArt, Life technologies, Regensburg, Germany) for recombinant expression in the *E. coli* platform. The sequence was cloned into pET22b (*amp*^*R*^) and transformed by heat shock in competent *E. coli* BL21 (DE3) cells.

*E. coli*/pET22b-HD5-GFP-H6-Cys was grown overnight at 37 °C and 250 rpm in Luria–Bertani (LB) medium with ampicillin at 100 μg/mL. The overnight culture was used as inoculum in shake flasks with fresh LB medium and ampicillin at 100 μg/mL starting at OD_600nm_ = 0.05. Cultures were grown at 37 °C and 250 rpm, and HD5-GFP-H6-Cys protein expression was induced by 1 mM IPTG when cultures reached an OD_600nm_ = 0.4–0.6. After 3 h, cultures were centrifuged at 6000 × *g* for 15 min at 4 °C, and the pellet was stored at −80 °C.

For protein purification, pellets were resuspended in binding buffer (500 mM NaCl, 20 mM Tris, 20 mM imidazole) with protease inhibitor and disrupted as described in ref (43). Supernatant obtained was further purified by Immobilized Metal Affinity Chromatography (IMAC) in an ÄKTA Start (GE Healthcare Bio-Sciences AB, Uppsala, Sweden) as described in ref (43).

### 4.4 TPU Surface Functionalization

The TPU discs were sonicated with isopropyl alcohol for 15 min. Then, the TPU surfaces were dried under a gentle N_2_ flow. After drying, the modification of the surface of the TPU was done in a three-step procedure. All steps were carried out under argon atmosphere, using dried toluene as solvent (and swelling agent) and dibutyltin dilaurate 95% as the catalyst. In the first step, the surface of the TPU discs was activated with HDI. Specifically, TPU samples were immersed into 5 mL of toluene containing 5% (v/v) HDI and 0.25% (v/v) dibutyltin dilaurate 95% for 1 h at 70 °C. Then, the TPU samples were washed with dried toluene for 30 min. In the second step, (i) PEG4K or (ii) PEG4K and Mal-PEG5K-OH were grafted onto the TPU surface by letting the hydroxyl groups react with the isocyanate end groups of the activated surface to obtain the surface-induced TPU-PEG assembled monolayer and the TPU-PEG-Mal mixed assembled monolayer, respectively. Specifically, the TPU surfaces were immersed in a 10% (w/v) PEG4K solution or a mixed solution of 40% Mal-PEG5K-OH and 60% PEG4K in dried toluene and left to react overnight at 45 °C. After that, the samples were washed in order to remove all the unreacted monomer with toluene, isopropyl alcohol, and Milli-Q water for 15 min each. The TPU discs were finally dried in a vacuum oven at 45 °C overnight to remove all solvents and restore their original size after swelling.

### 4.5 Bioclickable Protein Anchoring

The protein was anchored via a click reaction between the thiol of the cysteine group of HD5 (HD5-GFP-H6-Cys) and the maleimide terminated group of the surface-induced PEG assembled monolayer. Specifically, 80 μL of a 10 μM protein solution was deposited on top of the TPU surfaces that were functionalized with a mixed surface-induced assembled monolayer of PEG4K and Mal-PEG5K-OH and left to react in a wet chamber for 2 h at room temperature. Then, the substrates were rinsed 3 times with Milli-Q water and dried under a gentle N_2_ flow.

### 4.6 Instruments Used for the Characterization

The functionalized TPU discs were characterized using a multitechnique approach comprising FTIR, WCA, XPS, AFM, and a microplate reader for luminescence. FTIR spectra were obtained in a spectrophotometer, Jasco 4700, using an Attenuated Total Reflectance accessory. Resolution was 2, and the number of scans was 32. WCA measurement was performed at room temperature in a Drop Shape Analyzer, DSA 100, instrument from KRÜSS. WCA values of the samples were evaluated by static contact angle measurements using the sessile drop method. XPS measurements were performed at room temperature with a SPECS PHOIBOS 150 hemispherical analyzer (SPECS GmbH, Berlin, Germany)) at a base pressure of 5 × 10^–10^ mbar using monochromatic Al Kα radiation (1486.74 eV) as excitation source operated at 300 W. The energy resolution as measured by the fwhm of the Ag 3d_5/2_ peak for a sputtered silver foil was 0.62 eV. Charge neutralization was done through a flood gun. AFM characterization was performed using a Keysight 5100 AFM in tapping mode with an AppNano FORT tip under ambient conditions. SEM images were taken on a Quanta 650 FEG microscope at 5 kV. The luminescence experiment was measured at 520 nm at room temperature using the LUMIstar Omega microplate reader (BMG Labtech).

### 4.7 Antimicrobial Activity in Biofilm

To evaluate antimicrobial activity of the functionalized TPU surfaces, the strains selected were methicillin-resistant *S. aureus* ATCC-33592 (MRSA), *S. epidermidis* ATCC-35984 (MRSE), and *P. aeruginosa* ATCC-10145. MRSA, MRSE, and *P. aeruginosa* were grown in Brain Heart Infusion (BHI) broth (Scharlau, Barcelona, Spain), and tryptic soy broth (TSB) medium was used for biofilm formation.

An overnight culture of the selected strains was reinoculated in 10 mL of fresh BHI broth and grown at 250 rpm and 37 °C. Then a 1/200 dilution was done in TSB with the addition of 1% and 0.25% glucose for MRSA and MRSE, respectively. The TPU discs (functionalized or not with HD5-GFP-H6-Cys) were added by forceps to a 48-well plate, and a total of 250 μL/well of pathogen or control media was added. After 24 h of static incubation at 37 °C, the remaining liquid was removed with a vacuum pump, and wells were washed with 3 rounds of 300 μL/well of 0.9% NaCl (Ringer). A total of 400 μL/well of Ringer was added, and the plate was covered with parafilm to avoid volume loss and sonicated with the ultrasonic bath for 1 min, left in the refrigerator for 1 min, and sonicated again for 1 min. The content of the sonicated wells was transferred to eppendorf tubes and centrifuged at 6200 × *g*, 15 min, and 4 °C. Supernatant was removed, and pellets were fixed with 400 μL of 100% methanol for 10 min. Then the methanol was removed, and pellets were left to dry for 5 min more. Staining with 1% crystal violet (CV) in ddH_2_O was performed (400 μL/Eppendorf) for 20 min at room temperature. At the end of incubation, tubes were washed 3 rounds with 500 μL/Eppendorf ddH_2_O, and stained pellets were dissolved with 100 μL of 70% ethanol. Finally, the content of the tubes was transferred to a non-maxisorp plate and read at 595 nm in a microplate reader.

## Supporting Information Available

Basic chemistry of Thermoplastic Polyurethane (TPU); Stability test with Fourier transform infrared (FTIR) characterization; Stability test with Water Contact Angle (WCA) characterization; X-ray photoelectron spectroscopy (XPS) of the oxygen (O 1s); Atomic Force Microscopy (AFM) profiles, and Fluorescence plate reader images to optimize the protein anchoring step and the homogeneity of the functionalization (PDF)
