# Supplementary material for: Activating Thermoplastic Polyurethane Surfaces with Poly(ethylene glycol)-Based Recombinant Human α-Defensin 5 Monolayers for Antibiofilm Activity
Source: ACS Appl Bio Mater. 2025 Feb 20;8(3):1900–8. doi: 10.1021/acsabm.4c00732 (PMC11921020; doi:10.1021/acsabm.4c00732)
Supplement: Supplementary file 1 — mt4c00732_si_001.pdf [file mt4c00732_si_001.pdf]

Supporting Information

Activating Thermoplastic Polyurethane Surfaces with Polyethylene Glycol-Based Recombinant Human α-Defensin 5 Monolayers for Antibiofilm Activity

Xavier Rodríguez Rodríguez,[1,2] Adrià López-Cano,[3] Karla Mayolo-Deloisa,[1,4] Oscar Q Pich,[5,6] Paula Bierge,[5,6] Nora Ventosa,[1,2] Cristina García-de-la-Maria,[7] José M. Miró,[7,8] Oriol Gasch,[9] Jaume Veciana,[1,2] Judith Guasch,[1,2,10] Anna Arís,[3] Elena Garcia-Fruitós,[3] Imma Ratera[1,2,] and the FUNCATH investigators[11]*

[1] Institute of Materials Science of Barcelona (ICMAB-CSIC), Campus UAB, Bellaterra, 08193, Spain.

[2] Networking Research Center on Bioengineering, Biomaterials and Nanomedicine (CIBER-BBN), Campus UAB, Bellaterra, 08193, Spain.

[3] IRTA, Ruminant Production, Torre Marimon, Ctra C-59, Km 12,1, 08140 Caldes de Montbui, Barcelona, Spain

[4] Tecnológico de Monterrey, Institute for Obesity Research, School of Engineering and Sciences, Av. Eugenio Garza Sada 2001, 64849 Monterrey, Nuevo León, México.

[5] Laboratori de Recerca en Microbiologia i Malalties Infeccioses, Hospital Universitari Parc Taulí, Institut d'Investigació i Innovació Parc Taulí (I3PT-CERCA), Universitat Autònoma de Barcelona, 08208, Sabadell, Spain.

[6] Institut de Biotecnologia i Biomedicina, Universitat Autònoma de Barcelona, 08193 Bellaterra, Spain.

[7] Infectious Diseases Service. Hospital Clinic-FCRB-IDIBAPS. Universitat de Barcelona, Barcelona, Spain.

[8] Infectious Diseases Biomedical Research Networking Center (CIBERINFEC), Instituto de Salud Carlos III, Madrid, Spain.

[9] Servei de Malalties Infeccioses, Hospital Universitari Parc Taulí, Institut d'Investigació i Innovació Parc Taulí (I3PT-CERCA), Universitat Autònoma de Barcelona, 08208, Sabadell (Spain)

[10] Dynamic Biomimetics for Cancer Immunotherapy, Max Planck Partner Group, ICMAB-CSIC, Campus UAB, Bellaterra, 08193, Spain.

[11] FUNCATH Investigators are indicated in the author contributions

1. Basic chemistry of Thermoplastic Polyurethane (TPU)
2. Stability test with Fourier transform infrared (FTIR) characterization
3. Stability test with Water Contact Angle (WCA) characterization
4. X-Ray photoelectron spectroscopy (XPS) of the oxygen (O1s)
5. Atomin Force Microscopy (AFM) profiles
6. Fluorescence plate reader images to optimize the protein anchoring step and the homogeneity of the functionalization.

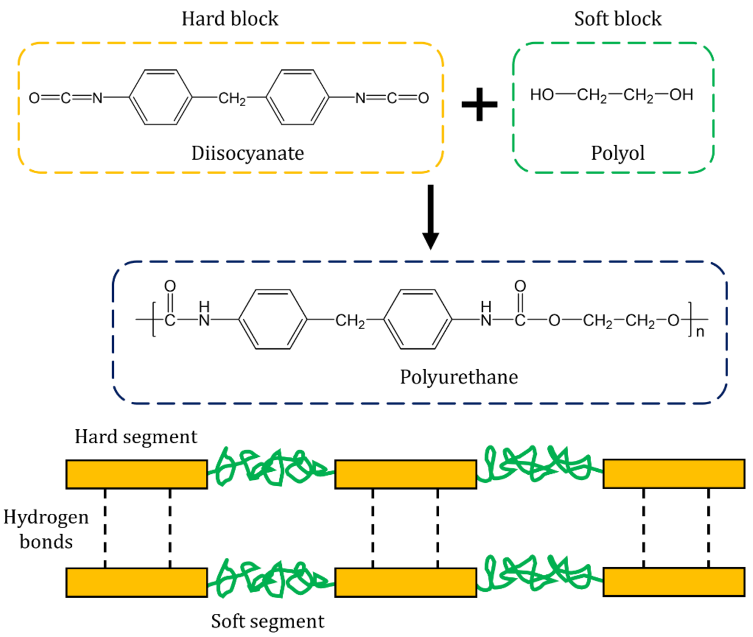

Figure S1. Basic chemistry of the TPU.

2. Stability test with FTIR characterization

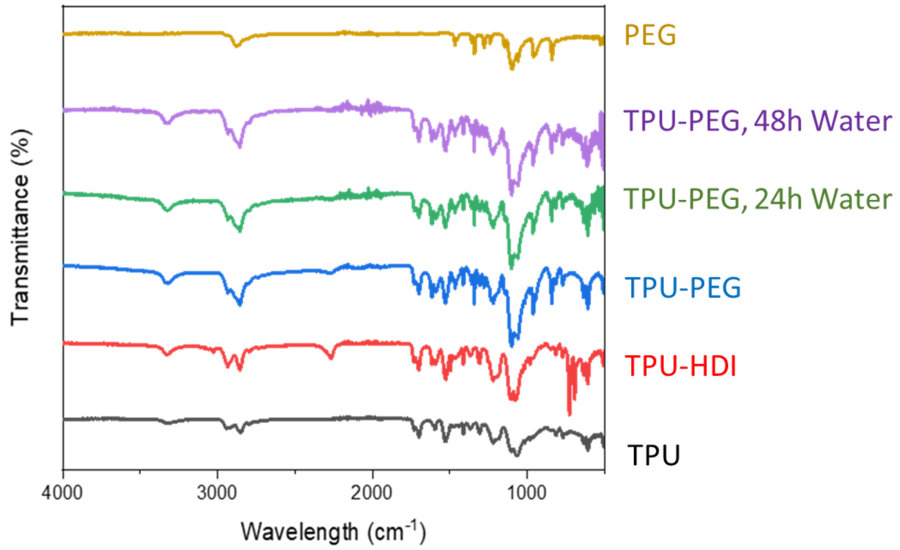

Figure S2. FTIR spectra were: (black) unmodified thermoplastic polyurethane (TPU); (red) functionalization of thermoplastic polyurethane with HDI (TPU-HDI); (blue) thermoplastic polyurethane with a PEG4K self-assembled monolayer (TPU-PEG); (green) thermoplastic polyurethane with a PEG4K self-assembled monolayer after 24h in water (TPU-PEG, 24h Water); (purple) thermoplastic polyurethane with a PEG 6000 self-assembled monolayer after 48h in water (TPU-PEG, 48 h Water); (yellow) PEG 6000 (PEG).

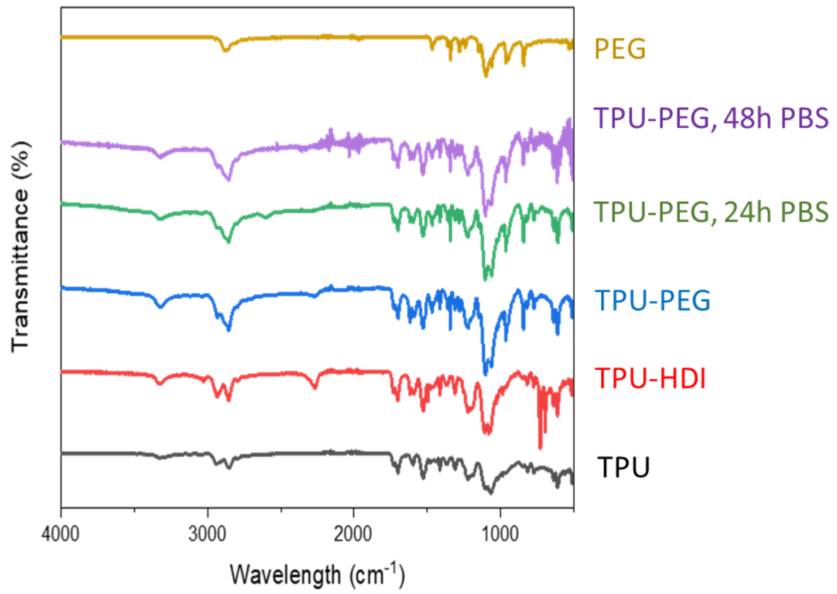

Figure S3. FTIR spectra were: (black) unmodified thermoplastic polyurethane (TPU); (red) functionalization of thermoplastic polyurethane with HDI (TPU-HDI); (blue) thermoplastic polyurethane with a PEG4K self-assembled monolayer (TPU-PEG); (green) thermoplastic polyurethane with a PEG4K self-assembled monolayer after 24h in PBS (TPU-PEG, 24h PBS); (purple) thermoplastic polyurethane with a PEG 6000 self-assembled monolayer after 48h in PBS (TPU-PEG, 48 h PBS); (yellow) PEG 6000 (PEG).

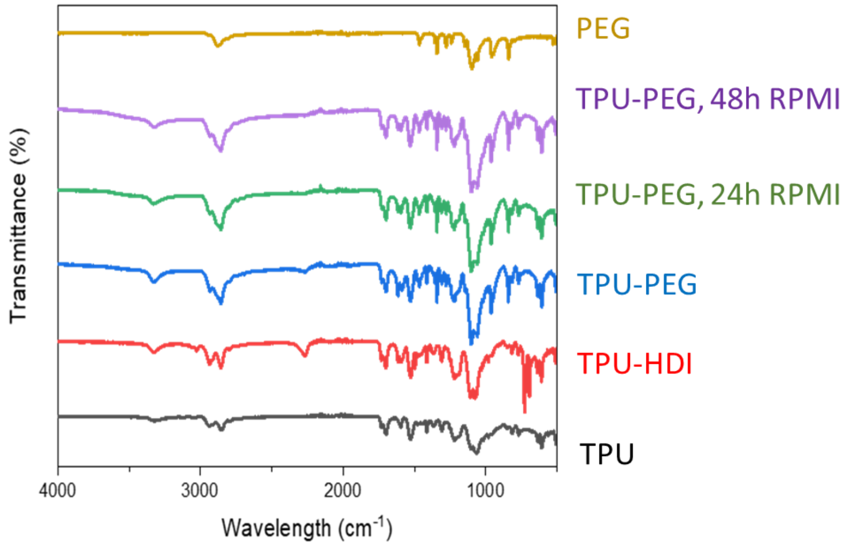

Figure S4. FTIR spectra were: (black) unmodified thermoplastic polyurethane (TPU); (red) functionalization of thermoplastic polyurethane with HDI (TPU-HDI); (blue) thermoplastic polyurethane with a PEG4K self-assembled monolayer (TPU-PEG); (green) thermoplastic polyurethane with a PEG4K self-assembled monolayer after 24h in RPMI media (TPU-PEG, 24h RPMI); (purple) thermoplastic polyurethane with a PEG 6000 self-assembled monolayer after 48h in RPMI media (TPU-PEG, 48 h RPMI); (yellow) PEG 6000 (PEG).

3. Stability test with WCA characterization

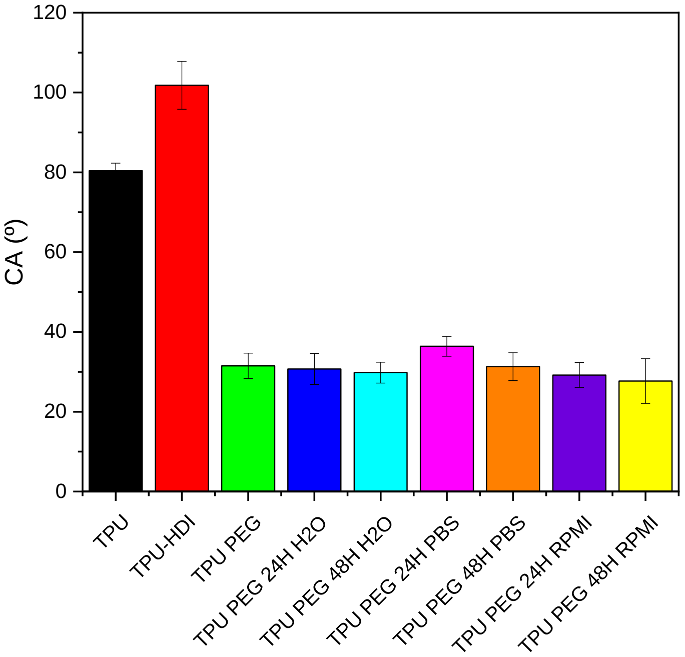

Figure S5. Water contact angle of: (black) the unmodified TPU; (red) functionalization of TPU with HDI; (green) thermoplastic polyurethane with a PEG4K self-assembled monolayer; thermoplastic polyurethane with a PEG4K self-assembled monolayer after (blue) 24h and (cyan) 48h in water, (magenta) 24h and (orange) 48h in PBS and (purple) and (purple) 24h and (yellow) 48h in RPMI media.

4. X-Ray photoelectron spectroscopy of oxygen (O1s)

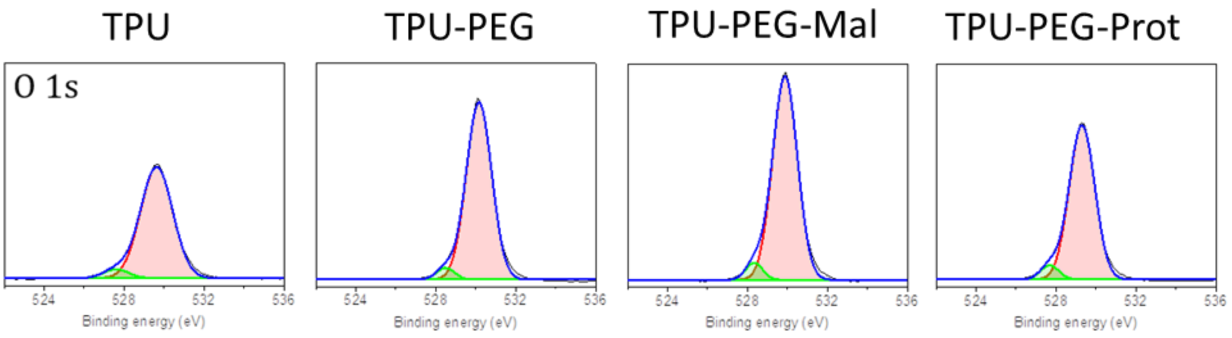

Figure S6. XPS spectra of O1s for unmodified TPU, TPU-PEG, TPU-PEG-Mal and TPU-Protein.

5. AFM profiles

TPU

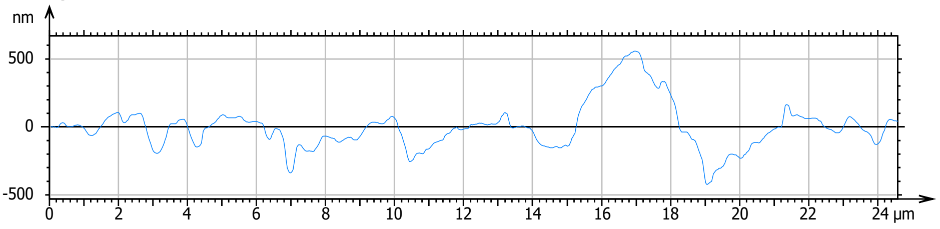

TPU-PEG

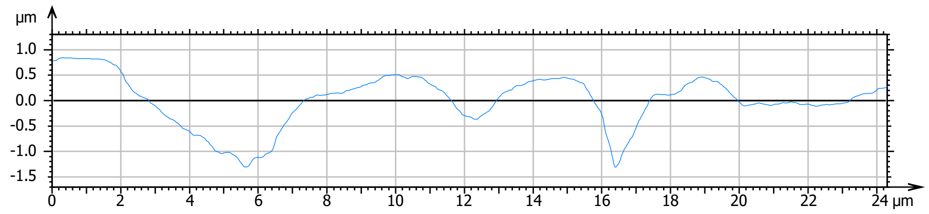

TPU-PEG-Mal

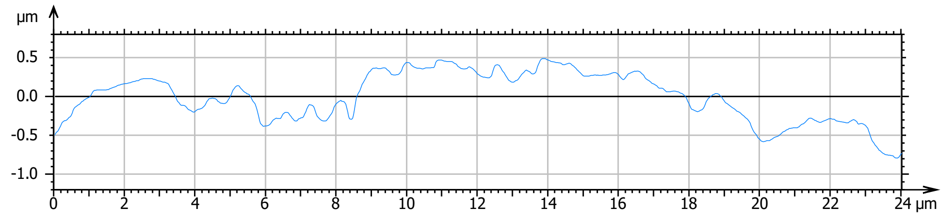

Figure S7. Profiles of the AFM images of: (top) unmodified thermoplastic polyurethane, (centre) thermoplastic polyurethane with a PEG 6000 surface-induced assembled monolayer and (bottom) thermoplastic polyurethane with a 40% maleimide terminated PEG and 60% PEG assembled monolayer.

6. Fluorescence plate reader images to optimize the protein anchoring step and the homogeneity of the functionalization.

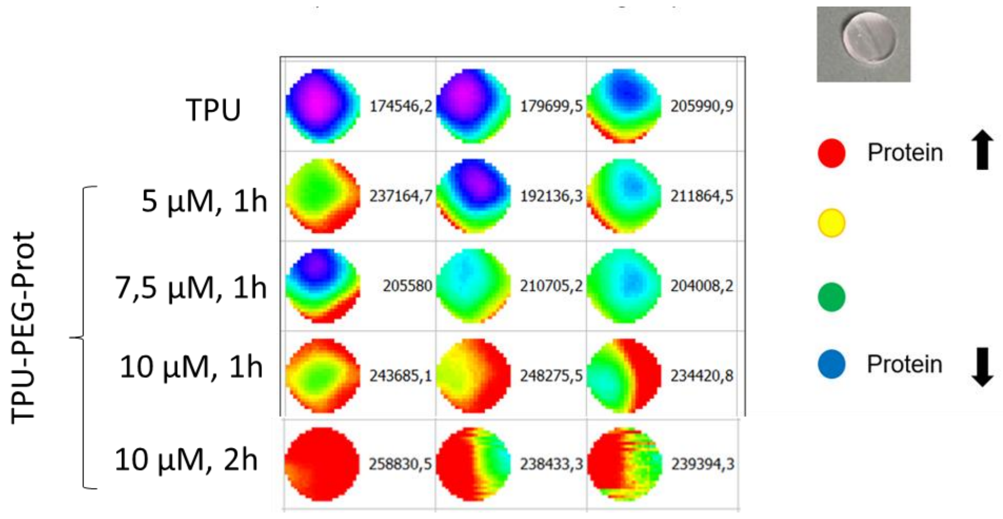

Figure S8. Fluorescence emission images using a microplate reader of the surface of the functionalized discs (PEG-Mal-prot) and non-functionalized TPU discs measured at 520 nm were the intensity of the fluorescence (which is proportional to the concentration of HD5-GFP-H6-Cys) is quantified as red > yellow > green > blue. Different protein concentrations and incubation times were analysed.
